# Molecular Characterizations of a Novel Putative DNA-Binding Protein LvDBP23 in Marine Shrimp *L. vannamei* Tissues and Molting Stages

**DOI:** 10.1371/journal.pone.0019959

**Published:** 2011-05-20

**Authors:** Yanisa Laoong-u-thai, Baoping Zhao, Amornrat Phongdara, Jinzeng Yang

**Affiliations:** 1 Department of Human Nutrition, Food and Animal Sciences, University of Hawaii at Manoa, Honolulu, Hawaii, United States of America; 2 Department of Chemical Engineering, Faculty of Engineering, Burapha University, Chonburi, Thailand; 3 Center for Genomics and Bioinformatics Research, Prince of Songkla University, Songkhla, Thailand; 4 College of Animal Science and Technology, Huazhong Agricultural University, Wuhan, People's Republic of China; University of South Florida, United States of America

## Abstract

**Background:**

*Litopenaeus Vannamei*, well known as pacific white shrimp, is the most popular shrimp in the world shrimp market. Identification and characterization of shrimp muscle regulatory genes are not only important for shrimp genetic improvement, but also facilitate comparative genomic tools for understanding of muscle development and regeneration.

**Methodology/Principal Findings:**

A novel mRNA encoding for a putative DNA-binding protein LvDBP23 was identified from *Litopenaeus vannamei* abdominal muscle cDNA library. The *LvDBP23* cDNA contains 639 nucleotides of protein-coding sequence with deduced 212 amino acids of predicted molecular mass 23.32 kDa with glycine-rich domain at amino acid position 94–130. The mRNA sequence is successfully used for producing LvDBP23 recombinant protein in sf9 insect cell expression system. The expression of *LvDBP23* mRNA is presented in abdominal muscle and swimming leg muscle, as well as other tissues including intestine, lymphoid and gill. The mRNA expression has the highest level in abdominal muscle in all tested tissues. LVDBP23 transcript during the molt cycle is highly expressed in the intermolt stage. *In vitro* nucleic acid-binding assays reveal that LvDBP23 protein can bind to both ssDNA and dsDNA, indicating its possible role of regulation of gene transcription.

**Conclusions/Significance:**

We are the first to report a DNA-binding protein identified from the abdominal muscle tissue of marine shrimp *L. Vannamei*. Its high-level specific expression during the intermot stage suggests its role in the regulation of muscle buildup during the growth phase of shrimp molt cycle.

## Introduction

*Litopenaeus Vannamei*, well known as pacific white shrimp, is the most popular shrimp in the world shrimp market [1]. Although the breeding programs for development of high-quality bloodstock have been widely carried out in several countries many shrimp researchers point out that the lack of genetic tools and understanding of molecular mechanisms of growth is still a hurdle for effective improvement of growth traits. A better understanding of muscle growth can have significant impacts on overall shrimp growth performances. Skeletal muscle is a remarkably plastic tissue. It can undergo dramatic changes in size and contractile properties during development, as well as when responding to a variety of physiological conditions. Differentiation of skeletal muscles begins when the mesodermal cells in the early embryo become attached to the myogenic lineage, which is then followed by the differentiation of fibers to specific types [2]. In mammals, this involves the expressions of skeletal muscle-specific transcription factors such as MyoD, MFY5, Myogenin, MRF4 and MEF2, which regulate the expressions of muscle-specific genes by interactions with the regulatory DNA sequences of targeted genes [2], [3]. After birth, a diverse number of factors such as hormones, active and passive stretch, use and disuse, and diseases can alter the size and fiber type composition of vertebrate skeletal muscles.

Crustacean muscle is structurally analogous to vertebrate skeletal muscles, with proteins organized in sarcomeres aligned along large penniform fibers. The main distinction of crustacean muscle is the different sarcomere length according to fiber type, with fast fibers organized in short sarcomeres and low mitochondrial density, and slow tonic fibers organized in long sarcomeres with high mitochondrial density. In crustaceans, muscle also exhibits a dynamic state of continuous atrophy and restoration to facilitate withdrawal from carapace at molting. The rate of *L. vannamei* growth rate varies with size, sex and time of year in the coastal waters. Molting frequency varies across different species, but is normally faster in early stages, slows down with age, and is strongly influenced by ecdsyteroid hormones [4]. Muscle loss during molting does not seem to occur in abdominal muscle [5]. Our results from SDS-PAGE analysis of abdominal muscles suggest the occurrence of muscle fiber rearrangement in both the premolt and postmolt stages [4].

The genes and molecular mechanisms of shrimp muscle growth have not received adequate scientific attentions to date. To gain a better understanding of shrimp growth and the underlying molecular mechanisms, we initiate a research project to identify muscle structural and regulatory genes by cDNA library and gene expression analysis. In a previous study, the abdominal muscle cDNA library of shrimp *L.vannamei* was successfully constructed [6]. Preliminary data analysis suggests that abundant and diverse transcripts are present in the cDNA library established by our laboratory. By degenerated PCR primer designing, we recently identified shrimp SUMO cDNA named LvSUMO-1 in *L. vannamei*. Expression of LvSUMO-1 mRNA suggest its role in the regulation of shrimp muscle protein degradation . In this research, we identified a novel gene encoding a putative DNA-binding protein, and further characterized its gene expression patterns in various shrimp muscle tissues and during the molt cycle.

## Results and Discussion

### LvDBP23 cDNA and Amino Acid Sequences analysis

**Figure 1:**
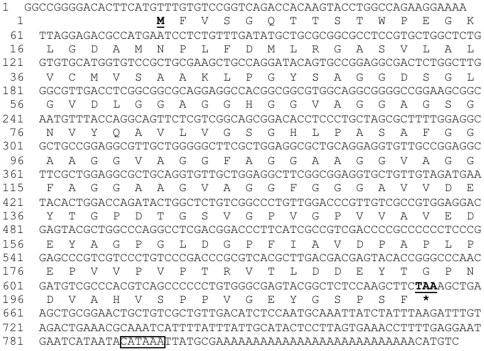
Nucleotide and deduced amino acid sequences of *LvDBP23* cDNA. Amino acids are indicated as single capital letters under each triplet codon of the nucleotide sequence. The start codon is the bold underline type and an asterisk (*) indicates the stop codon. The polyadenylation signal (CATAAA) is boxed.

**Figure 2:**
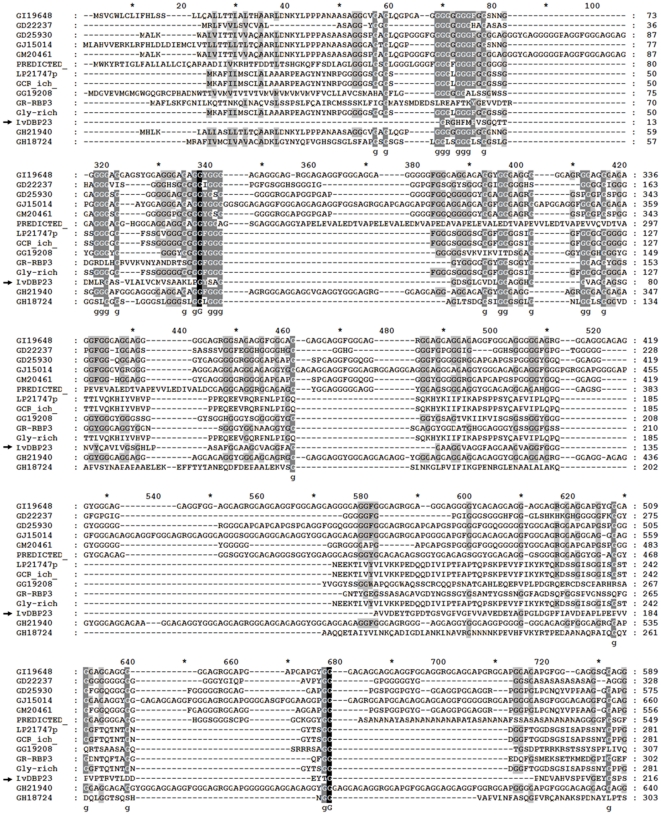
Sequence alignment of LvDBP23 with reported glycine-rich proteins. LvDBP23 amino acid sequence was aligned with reported glycine-rich proteins GI19648 (Accession No. XP_002004384), GD22237 (Accession No. XP_002079002), GD25930 (Accession No. XP_002081005), GJ15014 (Accession No. XP_002059851), GM20461 (Accession No. XP_002033356), PREDICTED: hypothetical protein (Accession No. XP_625289), LP21747p (Accession No. ABI34182), GCR(ich) CG5812-PA (Accession No. NP_651999), GG19208 (Accession No. XP_001977734), GR-RBP3 (Accession No. NP_200911), Gly-rich protein (Accession No. CAB66004), GH21940 (Accession No. XP_001987473), GH18724 (Accession No. XP_001989581).

By using degenerated oligonucleotide DNA probes, we have identified muscle regulatory genes from *L. vannamei muscle* cDNA library. One of the interesting genes reported here is a novel DNA binding protein named *LvDBP23*. This sequence partially matchs one of the probe sequences, but it does not have significant similarity to the gene that was used as a probe. To obtain a full-length mRNA sequence, we decided to carry out 5′RACE experiment. The result from 5′RACE DNA sequencing further extended 134 nucleotides at 5′ end from the primer sequence. The mRNA sequence of *LvDBP23* consists of 838 nucleotides, including an ORF of 639 nucleotides, 3′ UTR of 183 nucleotides with the stop codon (TAA) and polyadenylation signal of CATAAA sequence (Fig. 1). The mRNA sequence was submitted to NCBI GenBank (Accession number: JF742606). The deduced sequence of 212 amino acids from cDNA has a predicted molecular mass of 23.32 kDa. The BLAST result showed the similarity of LvDBP23 to glycine-rich protein of *Drosophila melanogaster* (Accession No. NP651999) with 44% identity in amino acid sequences. By protein alignment analysis of different glycine-rich proteins, it indicates that the glycine-rich region of LvDBP23 was presented on amino acid position 51–169 (Fig. 2.). In the motif scan bioinformatics software, the domain of Gly-rich is located in amino acid position 46–164 of LvDBP23 protein. The LvDBP23 amino acid sequence was also searched for conserved domain identifications. The Gly-rich domain is predicated to have DNA-binding function. Further online analysis indicates that DNA-binding site of LvDBP23 protein is located on the Gly-rich domain at the amino acid position 94–130. These data suggest that LvDBP23 is a putative DNA-binding protein.

### Production of LvDBP23 recombinant protein in SF9 insect cell line

**Figure 3:**
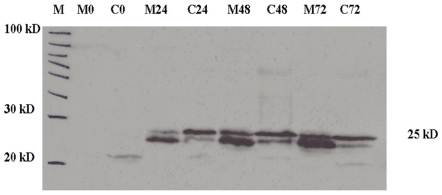
LvDBP23 protein expressions in Sf9 insect cell. The plasmids pIEx-5- *LvDBP23*-His was transfected to Sf9 insect cells. Transfected cells were incubated at 28°C for 0, 24, 48 and 72 hrs. Expressions of LvDBP23 were detected by Western blot analysis with anti-His.tag primary antibody. Lane M0, C0, M24, C24, M48, C48, M72 and C72 represent the protein extracts isolated from medium (M) or cellular (C) extracts of the indicated time of cell culture.

Compared with bacterial expression system, insect cell expression system is very useful for functional study of eukaryote protein because it can provide post-translational protein modification [7], [8]. To test the LvDBP23 mRNA sequence for appropriate translation to a protein, we used SF9 insect cells for producing recombinant protein. *GFP* and *LvDBP23* cDNA were inserted to pIEx-5 expression vector. Successful transfection of the plasmids was confirmed by observing green fluorescent signal with GFP plasmid with phase contract spectroscopy at 0, 24, 48, 72 hr after transfection. LvDBP23 protein was detected by western blot analysis at 25 kDa (LvDBP23-His) with anti-His antibody. The result shows the recombinant protein is presented in medium and cell fraction. The recombinant proteins apparently are made of two bands (Fig. 3). The sf9 cell expression system used in the study produce secretary recombinant proteins. The cell extracts have two bands with more the large-size protein while the extracts from the medium produce two bands with more the small-size proteins. Therefore, we believe the two bands are the large-size protein with the signal peptide predominantly in cell extract, and the small-size protein without the signal peptide predominantly in the medium. The protein levels appear to increase dramatically from 0 to 72 hrs of cell culture (Fig. 3).

### Expression of LvDBP23 gene in shrimp tissues

**Figure 4:**
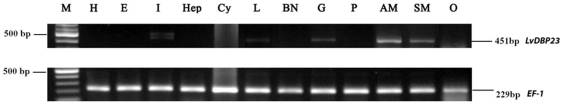
Tissue specificity of *LvDBP23* gene expression by RT-PCR. *LvDBP23* mRNA was detected in abdominal muscle (AM) and swimming leg muscle (SM) but weakly in intestine (I), lymphoid (L) and Gill (G). *EF-1* gene was used as a control gene. Abbreviations in the picture represented marker (M), heart (H), eyestalk (E), intestine (I), hepatopancreas (Hep), haemocyte (Cy), lymphoid (L), brain and nerve ganglion (BN), gill (G), pleopods (P), abdominal muscle (AM), swimming leg muscle (SM) and ovary (O).

**Figure 5:**
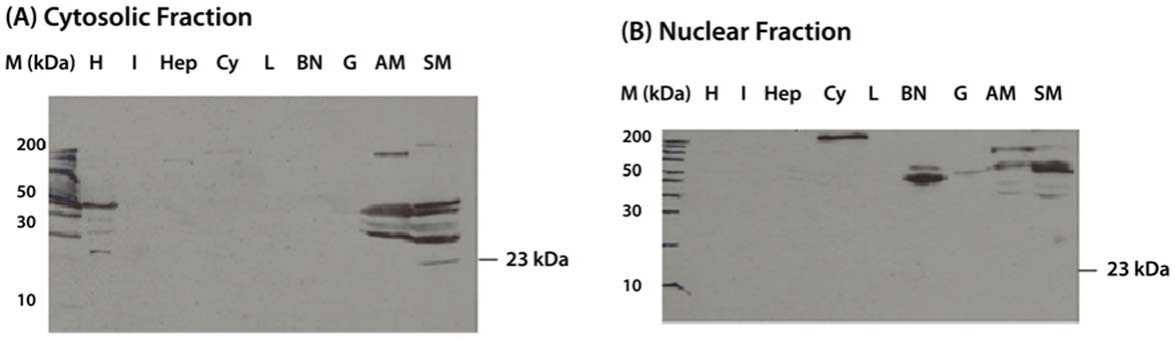
Tissue specificity expression of LvDBP23 protein by Western blotting. Detections of LvDBP23 protein in different tissues of cytosolic (A) and nuclear (B) fractions were shown including heart (H), intestine (I), hepatopancreas (Hep), haemocyte (Cy), lymphoid (L), brain and nerve ganglion (BN), gill (G), abdominal muscle (AM) and swimming leg muscle (SM). Shrimp tissues were collected from 3-month juvenile shrimp. M represents the protein marker in kDa. LvDBP23 protein was detected by polyclonal primary antibody to LvDBP23 specific 15-amino acids peptide.

To verify the expression of LvDBP23 transcripts and protein, we analyze the mRNA levels of LvDBP23 in various tissues from juvenile shrimp by reverse transcriptase-PCR. As shown in Fig. 4, the mRNA expressions were detected in intestine (I), lymphoid (L), gill (G), abdominal muscle (AM) and swimming leg muscle (SM) with the highest level in abdominal muscle. We also performed the protein expression by western blot analysis. The cytosolic and nuclear proteins were extracted and detected with LvDBP23 polyclonal antibody, which was customer-produced by Genscript. LvDBP23 protein was detected in the cytosolic fraction of the swimming leg muscle tissue (SM) at the molecular size 23 kDa (Fig. 5). These results suggested that the LvDBP23 is not a muscle-specific protein, but expressed in several other types of shrimp tissues. LvDBP23 protein was not detected in AM and other tissue samples. One possible reason may be due to the protein composition in AM samples, which may have a low percentage of the target protein (LvDBP23), but a large percentages of structural proteins such as actin, myosin etc. it may also be caused by a high degree of post-translational modifications in the tissues used in the Western blot analysis. The exact reasons of undetectable levels of LvDBP23 protein in these tissues deserve further investigations.

### LvDBP23 expression during molting cycle

**Figure 6:**
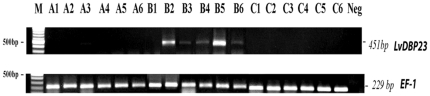
*LvDBP23* gene expression in different molting stages by RT-PCR. Six individual shrimp of each stages of postmolt (A), intermolt (B), premolt (C) were used for LvDBP23 gene expression analysis. Reverse transcription PCR of LvDBP23 partial mRNA sequence (451 bp) was carried out with mRNA isolated from shrimp abdominal muscle. EF-1 gene was used as a control gene.

The *LvDBP23* gene was originally identified from shrimp muscle cDNA library and had the highest expression in abdominal muscle tissue. We performed RT-PCR to study *LvDBP23* gene expression in shrimp abdominal muscle during different stages of molting cycle. The result showed that *LvDBP23* expression level was highly up-regulated in the intermolt stage (Fig. 6). Earlier reports indicate that there is high muscle protein synthesis during intermolt [5], [9]. A high level of *LvDBP23* in the intermolt stage suggests that LvDBP23 may involve in the regulation of shrimp muscle protein synthesis.

Net muscle gain during a molt cycle is the result of protein synthesis and degradation. Although factors that control and influence both protein synthesis and degradation are critical for understanding shrimp growth, there is very limited research data available for shrimp species. The muscle morphology and biochemical changes of *L. vannamei* during molting process have been studied by Yang and his research group [4], [10]. They found that the muscle structural α-actin and cytoskeleton β-actin were increased during the intermolt stages, suggesting the high muscle growth during these stages. Another protein named LvSUMO-1 has been reported as the regulatory protein in shrimp myogenic differentiation and muscle formation [11]. We have not specifically identified muscle regulatory proteins well studied in vertebrate animals from this shrimp cDNA library. Several myogenic regulatory factor (MRF) proteins expressed during myoblast cell differentiation such as MyoD, myogenin, Myf5, and MRF4 have also been well characterized in model animals [2]. The MyoD gene family has been shown to be conserved from Drosophila to vertebrates. In the invertebrates, one type of MRF family members was identified in the species *C. elegans*, sea urchin and Drosophila. The nautilus protein (NAU) was identified in Drosophila, it has a typical feature of MRF protein with a bHLH domain and has 90% similarity to the vertebrate MRF members [12]. *LvDBP23* is not grouped to any these muscle regulatory proteins or protein family.

### In vitro nucleic acid-binding assays of LvDBP23

**Figure 7:**
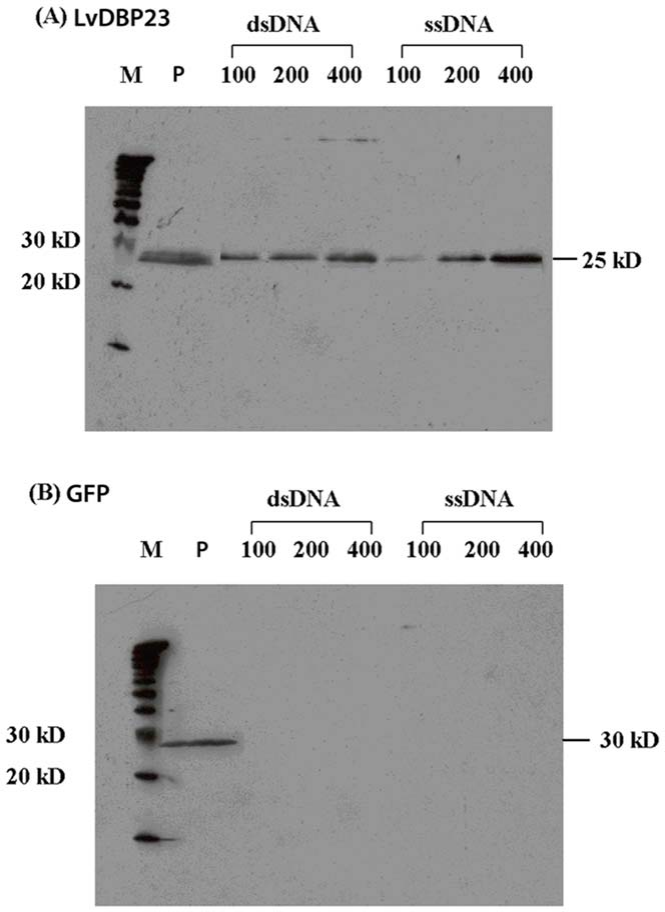
DNA binding protein assay. LvDBP23 (A) and GFP (B) proteins binding with dsDNA cellulose and ssDNA cellulose bead in different total protein amount of 100, 200, 400 µg. P: only recombinant protein without DNA in the reaction. Both proteins were detected by anti-His antibody.

Different amounts of total secreted recombinant protein of GFP-His (control) and LvDBP23-His were incubated with calf thymus ssDNA-cellulose and dsDNA-cellulose beads. The result showed that only LvDBP23 protein could bind with both ssDNA and dsDNA (Fig. 7). The binding efficiency was highly dependent on the amount of protein. LvDBP23 protein showed higher interaction with ssDNA than dsDNA (Fig. 7A). In vitro nucleic acid-binding assay had been used to identify DNA/RNA binding proteins such as GR-RBP4 , DdrB, ribonucleoprotein and SSB [13]–[16]. Based on the earlier Gly-rich domain prediction (94–130 amino acid), the results from DNA binding assays clearly suggest that LvDBP23 is a putative DNA-binding protein. According to the mRNA expression of LvDBP23 during molt cycle clearly showed the expression only in intermolt, whose stage is characterized by high muscle protein synthesis [9], [12]. This may indicate that LvDBP23 may act as transcription factor regulating the muscle protein gene expression. Further experiments in identifying the specific DNA sequence with which LvDBP23 interacts will help classify its protein nature and function.

Several transcription factors have been reported to consist of Gly-rich amino acid region. For example, transcription factor NF-kB is originally identified as a transcription factor that binds to the kB site in the intronic enhancer of the immunoglobulin k light-chain gene in B lymphocytes [17]. The Gly-rich region in NF-kB p105 functions as a processing signal for the generation of the p50 subunit [18]. In Drosophila, a DNA binding protein named MSL1 also contains a glycine-rich motif between the basic and leucine-zipper-like motifs, which mediates MSL1 self-association in vitro and binding of the amino-terminal region of MSL1 to the MSL complex assembled on the male X chromosome. Its basic region may mediate DNA binding, and the glycine-rich region may promote the association of MSL1 complexes to closely adjacent sites on the X chromosome [19]. However, glycine-rich proteins in plant can act as RNA binding proteins. In vitro nucleic acid-binding assays revealed the glycine-rich RNA-binding protein4 (GR-RBP4) binding sequence interacts with non-specifically to RNAs and DNAs [13]. Proteins that contain RNA-binding proteins (RBPs) and a glycine-rich region at the C-terminus (glycine-rich RNA-binding proteins, GR-RBPs) have been described in plants, and their involvement in plant stress response has been indicated by several expression analyses in maize [20].

In conclusion, we report a new shrimp mRNA named *LvDBP23* identified from *L vannamei* abdominal muscle cDNA library. The 838 nucleotides of *LvDBP23* cDNA contain a protein-coding sequence of 639 nucleotides and 183 nucleotides of 3′ non-coding region with the stop codon (TAA) and polyadenylation signal (CATAAA). This *LvDBP23* mRNA was highly expressed in abdominal muscle of the juvenile shrimp, along with other tissues such as swimming legs, intestine, lymphoid and gill. The mRNA sequence of LvDBP23 was successfully used for recombinant protein production in insect cell culture. *LvDBP23* mRNA has a high level of expression during the intermot stage. Nuclei acid-binding assay showed LvDBP23 has both ssDNA and dsDNA binding properties, which support the predicted function of the DNA binding Gly-rich domain (amino acid 94–130). Therefore, we concluded that LvDBP23 is a novel putative DNA-bind protein, which may involve in regulation of muscle protein gene expression during the intermolt stage.

## Materials and Methods

### Animals and tissue sampling

Cultured, specific pathogens free (SPF) nauplius, postlarvae and 3-months-old *Litopenaeus vannamei* from a local shrimp farm in Kahuku HI were transported alive in plastic bags with 1/3 water and 2/3 compressed air to the laboratory. Fresh tissues were collected for RNA and protein extraction following the procedure outlined by Laoong-u-thai et al. [11]. The tissue samples used for molting stage studies were the same lot as in previous reports [10], [11].

### cDNA identification and sequence analysis

Previously, we have described the *L. vannamei* muscle cDNA library [6]. Positive muscle cDNA clones were identified by using phage lift hybridization method as described by [11]). To further identify regulatory genes for muscle growth. We believe that TGF-β proteins and muscle transcription factor play critical roles in control muscle formation and growth. Therefore, six degenerated oligonucleotide DNA probes designed by using the conserved sequences of TGF-β and muscle transcription factor (myostatin, myostatin-like genes, MyoD and myogenin) of vertebrate and invertebrate species, including *H. sapiens*, *P. troglodytes*, *C. familiaris*, *M. musculus*, *R. norvegicus*, *G. gallus*, *D. melanogaster*, *A. gambiae* and *A. irradians*. These oligonucleotide DNA probes were labeled with biotin (IDT, Coralville, IA). Hybridization was carried out as described [11]. After hybridization, the filters were washed in 2× SSC, 0.1% SDS two times for 15 min at room temperature and once for 15 min at 50°C of 0.1× SSC, 0.1%SDS. Blots were autoradiographed for empirically optimized exposure times by using CDP-Star® Chemiluminescence reagent as substrate for streptavidin alkaline phosphatase conjugates. The nucleotide and protein sequence similarities searches were conducted with BLAST algorithm at the National Center for Biotechnology Information (NCBI, http://www.ncbi.nlm.nih.gov/BLAST/).

To get full-length cDNA sequences, we also did 5′RACE experiment. The extension of 5′ sequence was amplified by 5′ RACE GeneRacer system (Invitrogen) with shrimp muscle mRNA template and *LvDBP23* specific primer (GSP: 5′ CCGCTTCCGGCCCCGC CTGCCACGC 3′) and nested specific primer (Nested GSP: 5′CTGTATCCTGGC AGCTTCGCAGCGG 3′). Abdominal muscle mRNA was dephosphorylated by calf intestinal phosphatase (CIP) to eliminate truncated mRNA and other RNA. Dephospholylated mRNA was treated with tobacco acid pyrophosphatase (TAP) to remove 5′cap structure from full-length mRNA. Treated mRNA was ligated with GeneRacer™ RNA oligo to 5′end of mRNA using T4 RNA ligase. A cDNA template was generated by reverse transcription using SuperScript® III and random primers. Firstly, the 5′ end cDNA sequence was amplified with the appropriate GeneRacer™ 5′ Primer and a gene-specific primer. Nested PCR was performed by using GeneRacer™ 5′ nested Primer and a nested gene-specific primer.

ORF finder (NCBI) program was used for prediction of start codon position. The deduced amino acid sequence was analyzed with the NCBI Protein Sequence Analysis software. Predictions of protein function were initially analyzed by online software named Motif Scan (http://hits.isb-sib.ch/cgi-bin/PFSCAN). Search for conserved domains were analyzed on NCBI website (http://www.ncbi.nlm.nih.gov/Structure/cdd/wrpsb.cgi). DP-Bind, a web server for sequence-based prediction of DNA-binding residues, was used for searching DNA binding sequences (http://lcg.rit.albany.edu/dp-bind/).

### Expression of LvDBP23 recombinant protein in SF9 insect cell line

The Insect cell expression (Novegen, Gibbstown, NJ) system was used to express recombinant *LvDBP23* protein. Briefly, *LvDBP23* and *GFP* gene were inserted to pIEx-5 plasmid between Nco *I* and Xho *I* restriction site. This plasmid has the protein secretion property. The plasmids pIEx-5- *LvDBP23*-His and pIEx-*GFP*-His were transformed into bacterial strain One Shot® TOP10. Plasmids were prepared by GeneJET™ Plasmid Miniprep Kit (Fermentas, Glen Burnie, MD), and inserts were confirmed by sequencing. The plasmids were transfected into *Sf9* cells. Monolayer of Sf9 cells was grown in T-75 flasks at 28°C containing 9 ml of BacVector Insect cell medium supplement with 5% (v/v) fetal bovine serum by typical seeding density 1.0×10^6^ cell. Cell density was determined by hemacytometer counts and cell viability was evaluated by Trypan Blue exclusion dye at 0.4% (w/v) in 0.85% phosphate buffer saline (PBS). In T-75 flask , monolayers adherent Sf9 cells diluted with 10 ml serum free BacVector Insect cell medium with cell density 4×10^5^ cell/ml were transfected with 8 µg pIEx5-*LvDBP23* and pIEx5-*GFP* which were diluted with 400 µl serum free BacVector Insect cell medium, using 40 µl GeneJuice Transfection Reagent (Novegen, Gibbstown, NJ), according to the manufacturer's protocol. Transfectants were incubated at 28°C for 48 hrs. Transfected cells were lysed by 50 µl Insect PopCulture® Reagent per 1 ml culture volume, followed by 10 U Benzonase® nuclease per 1 ml of the original culture volume [21]. The mixture was gently inverted several times and incubates 15 min at room temperature. Expression of LvDBP23-His was monitored by SDS-PAGE and western blot analysis with anti-His.tag primary antibody.

### RNA extraction

Fresh different tissues of juvenile shrimp *L. vannamei* including heart (H), eyestalk (E), intestine (I), hepatopancreas (Hep), haemocyte (Cy), lymphoid (L), brain and nerve ganglion (BN), gill (G), pleopods (P), abdominal muscle (AM), swimming leg muscle (SM) and ovary (O) and muscle tissues of different developmental stages including nauplius , postlarva and juvenile were collected for RNA extraction. Total RNA was isolated by TRIZOL Reagent (Invitrogen, Carlsbad, CA), following the method described of previous work [22]. Total RNA concentrations were determined by a spectrophotometer [23].

### Protein extraction

Cytoplasmic and nuclear protein were extracted as previously described [11]. Briefly, 150 mg tissue was homogenized in buffer A (10 mM HEPES, pH 7.9, 10 mM KCl, 0.1 mM EDTA) contained protease inhibitor (1 mM DTT, 0.5 mM PMSF, 10 µg/µl leupeptin) and 40 µl of 10% NP40, incubated at room temperature. Cell debases and un-break cell was separated by centrifugation follow by another centrifugation for nuclear and cytosolic protein separation. Nuclear pellet was resuspened by buffer B (20 mM HEPES, pH 7.9, 0.4 M NaCl, 1 mM EDTA, 10% glycerol) as the nuclear protein fraction. Protein concentration was determined by using BCA method (Pierce).

### Expression of LvDBP23 in different tissues

Total RNA samples were treated with DNaseI to degrade any possible genomic DNA. Briefly, total RNA (1.5 µg) was added in a 10 µl reaction containing 1 µl 10× buffer (200 mM Tris-HCl, pH 8.4, 20 mM MgCl2, 500 mM KCl), 1.5 µl DNase I (1.5 U, Invitrogen, Carlsbad, CA) at 37°C for 15 min, followed by inactivation with 25 mM EDTA at 65°C for 10 min. Reverse transcription (RT) was performed using 1.5 µg RNA, 0.5 µg Oligo (dT) 18 mers, 1.0 mM dNTP, 4 µl of 5× reaction buffer and 40 U of M-MuLV reverse transcriptase (Fermentas, Burlington, Canada) at 37°C for 60 min. The reaction was stopped by heating at 70°C for 10 min. For PCR amplification, two oligonucleotide primers, B47_F2: 5′ GCGACTCTGGCTTGGGCGTTGAC 3′ and B47_R2: 5′CTGACGTGGGCGACATCGTTG GG 3′ were designed based on the nucleotide sequence corresponding to the nucleotide positions the position 168–190 (B47_F2) and 609–635 (B47_R2) of *L. vannamei LvDBP23* cDNA, respectively.

A set of control, one pair of elongation factor 1α (*EF-1α*) primer, EF-1_F and EF-1_R [24] was used during the RT-PCR amplification. PCR reactions were conducted for 40 cycles with denature at 94°C for 30 sec, annealing at 60°C for 30 sec, and extension at 72°C for 30 sec in a reaction buffer consisting of 10 mM Tris (pH 8.3), 50 mM KCl, 200 M dNTPs, 0.2 µM of each primer, and 2.5 U of Taq Polymerase (Biolabs Inc., Ipswich, MA) in a total volume of 50 µl. To visualize the amplified products, PCR reaction was electrophoresed through 1.5% agarose gel containing 0.025% ethidium bromide and observed under the UV light.

### Western blot analysis

The primary antibody were custom-produced by using the synthesized peptide of amino acid position 185–198 (CVTLDDEYTGPNDVA) of LvDBP23 protein, which was designed by online antigen software for good antigenicity and surface probability. The cytosolic and nuclear protein were subjected to electrophoresis using 15% SDS-PAGE, following by Western blotting according to the Mini Tran-Blot electrophoretic Transfer cell system (BioRad, Hercules, CA). Proteins were transferred onto PVDF membrane (BioRad, Hercules, CA) in electro blotting buffer (25 mM Tris–HCl, 190 mM Glycine, 20% methanol) at a constant current of 2.5 mA for 2 h. The membrane was immersed in blocking buffer (5% nonfat dry milk, 1× Tris buffer saline (TBS), pH 7.4, 0.1% Tween 20) at room temperature for 1 hr followed by incubation with primary antibody (rabbit polyclonal antibody, GenScript) in antibody dilution buffer (1% nonfat dry milk, 1× Tris buffer saline (TBS), pH 7.4, 0.1% Tween 20) at a concentration of 1∶500 at 4 C overnight. Subsequently, the membrane was incubated in HRP-conjugated anti-rabbit IgG (Santa Cruz) for 2 h at a concentration of 1∶1000, washed five time for 5 min 2 time, 10 min 2 time and 20 min 1 time with 0.1% tween-20, 1×TBS and developed with Visualizer EC western blot detection kit (Millipore/Upstate, Temecula, CA).

### In vitro DNA binding assay

The total secreted recombinant proteins (100, 200 and 400 µg) of LvDBP23-His and GFP-His were incubated with 5 µl of ssDNA-cellulose and dsDNA-cellulose beads (USB) at a concentration of 10 mg/ml in 20 µl of binding buffer (10 mM TRIS-HCl, pH 7.4, 2.5 mM MgCl2, 0.5% Triton X-100, and 125 mM NaCl and 10% Glycerol) with 1 µl each of 10 mg/ml Leupeptin and 1 mM Dithiothreitol (DTT). The mixture was incubated on ice at 4°C for overnight with shaking, and the beads were washed three times to remove the unbound-proteins with the 100 µl of binding buffer (without Leupeptin and DTT). After the last wash, the samples were re-suspended with 10 µl of binding buffer follow by boiling in SDS loading buffer. The amount of LvDBP23-His and GFP-His protein retained on the beads was determined by western blotting with anti-His antibody.
