# Correction: Molecular Characterizations of a Novel Putative DNA-Binding Protein LvDBP23 in Marine Shrimp *L. vannamei* Tissues and Molting Stages

**DOI:** 10.1371/annotation/491ecefd-50ca-4d44-9618-9c771a5ce1ca

**Published:** 2011-07-19

**Authors:** Yanisa Laoong-u-thai, Baoping Zhao, Amornrat Phongdara, Jinzeng Yang

The authors would like to add the following grant to their funding information: "Faculty of Engineering research grant (2010), Burapha University Thailand (Grant #33/2553)." 

